# Growing up in armed groups: trauma and aggression among child soldiers in DR Congo

**DOI:** 10.3402/ejpt.v4i0.21408

**Published:** 2013-11-06

**Authors:** Katharin Hermenau, Tobias Hecker, Anna Maedl, Maggie Schauer, Thomas Elbert

**Affiliations:** 1 Department of Psychology, University of Konstanz, Konstanz, Germany; 2 vivo international, Allensbach, Germany

**Keywords:** PTSD, child soldiers, aggression, violence, DRC

## Abstract

**Background:**

Child soldiers are often both victims and perpetrators of horrendous acts of violence. Research with former child soldiers has consistently shown that exposure to violence is linked to trauma-related disorders and that living in a violent environment is correlated with enhanced levels of aggression.

**Objective:**

To gain more insight into the experiences and the mental health status of former child soldiers, we conducted a survey with *N*=200 former child soldiers and adult combatants in the DR Congo.

**Methods:**

We conducted semi-structured interviews concerning military experiences, experienced and perpetrated violence, and mental health.

**Results:**

Former child soldiers reported more experienced and perpetrated violence, a greater severity of trauma-related suffering, as well as higher appetitive aggression than adult ex-combatants. Appetitive aggression was related to more perpetrated violence, higher military ranks, voluntary recruitment and higher rates of reenlistments in former child soldiers.

**Conclusions:**

Our results indicate that growing up in an armed group is related to higher levels of trauma-related disorders and aggressive behavior. This may explain the challenge of reintegrating former child soldiers. It is thus important to consider mental health problems, particularly trauma-related disorders and aggressive behavior, of former child soldiers for designing adequate reintegration programs.

The use of child soldiers is very common in current on-going conflicts worldwide (Elbert, Rockstroh, Kolassa, Schauer, & Neuner, 2006; Guy, 2009; Maedl, Schauer, Odenwald, & Elbert, 2010; Shaw, 2000). Hereby, child soldiers are defined as individuals under the age of 18 associated with armed forces (Coalition to stop the use of child soldiers, 2008; UNICEF, 1997, 2007). They do not only take part in combat but they also often work as carriers, guards, domestic servants, or sex slaves (Coalition to stop the use of child soldiers, 2010; Schauer & Elbert, 2010; UNICEF, 2007). Child soldiers are known to be involved in conflicts in at least 86 countries and territories worldwide (Coalition to stop the use of child soldiers, 2008). In the eastern provinces of the Democratic Republic of the Congo (DRC), recruitment of child soldiers is an entrenched feature of on-going armed conflict (Coalition to stop the use of child soldiers, 2008; Guy, 2009; United Nations, 2007). While local militia groups (e.g., Mai-Mai groups) and foreign armed groups (e.g., Forces démocratique pour la libération du Rwanda, FDLR) are known to recruit child soldiers, they are also present in the national army (Forces armées de la Republique démocratique du Congo, FARDC) (Coalition to stop the use of child soldiers, 2008; Davis & Hayner, 2009). In 2007, an estimated 7,000 child soldiers still remained in armed groups and forces mostly in eastern Congo. Despite new laws prohibiting the recruitment of children (defined in the DRC as under 18 years of age) and banning child soldiering (Coalition to stop the use of child soldiers, 2008, 2010; Guy, 2009), child recruitment by Mai-Mai groups, FDLR and Congès nationale du peuple (CNDP) continues to rise.

Armed groups in the DRC continue to forcefully abduct children (Coalition to stop the use of child soldiers, 2010; Romkema, 2007). In contrast, groups identifying themselves by their particular ethnic background recruit by emphasizing the need to defend their own people. Some minors join armed groups wishing to take revenge on the perceived enemy or hoping for a better life and more status (Coalition to stop the use of child soldiers, 2010).

As typical for “new wars” (Elbert et al., 2006; Shaw, 2000), child soldiers in the conflict in the eastern DRC begin their military career from the bottom, whereas adult combatants and soldiers start with higher ranks depending on their educational background or age. Frequently, child soldiers have to execute the most dangerous and gruesome tasks in which they experience and perpetrate significant amounts of violence (Pham, Vinck, & Stover, 2009; Schauer & Elbert, 2010). They suffer heavily from the consequences of being both victims and perpetrators in on-going conflicts (Betancourt, Simmons, Borisova, & Brewer, 2008; Derluyn, Broekaert, Schuyten, & De Temmerman, 2004; Schauer & Elbert, 2010; Stott, 2009). Exposure to severe and traumatic stress may lead to the development of posttraumatic stress disorder (PTSD). According to the “building block effect,” repeated exposure to different types of traumatic stressors cumulatively heightens the risk of developing trauma-related disorders like PTSD (Neuner et al., 2004; Schauer et al., 2003). Thus, child soldiers are highly vulnerable to developing psychological disorders. These psychological disorders have further effects on functionality, physical health, and mortality (Schauer & Elbert, 2010; Vinck, Pham, Stover, & Weinstein, 2007).

Living in a violent environment can additionally result in more aggressive behavior, particularly in men and boys who have had combat experience (Betancourt et al., 2010; Catani, Jacob, Schauer, Kohila, & Neuner, 2008; Schauer & Elbert, 2010). Persons suffering from PSTD become aroused easily and may respond aggressively to a perceived threat (Elbert et al., 2006; Maedl et al., 2010). In addition to reactive aggression, armed groups instrumentally act aggressively to achieve external goals such as obtaining ammunition, food, money, recruiting new soldiers or upholding their reputation. Former child soldiers reported that their experience of war brought about a gradual transformation in their perception of violence: At first it was frightening, but with repeated experience it became not only normal and acceptable, but even exciting and arousing (Elbert, Weierstall, & Schauer, 2010; Maclure & Denov, 2006). This appetitive form of aggression is conceptualized as perceiving aggressive behavior toward others as arousing and fascinating, even without gaining any immediate external benefit (Hecker, Hermenau, Maedl, Elbert, & Schauer, 2012). The phenomenon of “appetitive aggression” has only recently begun to receive attention. Research with former child soldiers in northern Uganda has shown that living in a violent environment is correlated with appetitive aggression (Elbert et al., 2010). Other studies with different samples in Uganda (Weierstall, Schalinski, Crombach, Hecker, & Elbert, 2012) and the DRC (Hecker et al., 2012) showed that appetitive aggression is also linked to higher rates of perpetrated violence. A study with Rwandan prisoners (Weierstall, Schaal, Schalinski, Dusingizemungu, & Elbert, 2011) found a protective effect of appetitive aggression on PTSD symptoms However, recent research showed that appetitive aggression protects against PTSD symptoms only if the level of traumatization does not exceed a certain threshold (Hecker, Hermenau, Maedl, Schauer, & Elbert, 2013; Weierstall, Bueno Castellanos, Neuner, & Elbert, 2013). Previously, the theory was further outlined that the inhibition of intra-species killing needs to be learned (Elbert et al., 2010). In a peaceful society, moral and social norms restrict extreme forms of violence. Life in an extremely violent environment, such as an armed group, can break down this socially learned inhibition (Engen, 2008). If the inhibition breaks down or is not learned, as is potentially the case for many young child soldiers, violence can be perceived appetitively. Furthermore, appetitive aggression has been shown to be positively related to holding higher military ranks in armed groups (Crombach, Weierstall, Hecker, Schalinski, & Elbert, 2013). However, research on the development of appetitive aggression is still lacking.

Besides a variety of challenges, for example, resource deficits in food, education, work, and psychological support (Maedl et al., 2010; Mogapi, 2004; Stott, 2009), mental health problems (e.g., PTSD symptoms) and a high tendency toward aggressive behavior—especially appetitively aggressive behavior—can pose a challenge to integration into communities (Betancourt et al., 2010; Elbert et al., 2010; Medeiros, 2007; Pham et al., 2009). Therefore, it is crucial to get more insight into the experiences and mental health problems of child soldiers in order to adjust reintegration programs to better meet the child soldiers’ needs and help them to integrate into a peaceful, civil society.

In this study, we aimed to examine the characteristics of former child soldiers in the DRC concerning their time and experiences in armed groups and the relation to their mental health status (i.e., PTSD symptom severity and appetitive aggression). Therefore, we compared them to former adult soldiers and combatants (below referred to as combatants) shortly after demobilization. Based on recent reports (Coalition to stop the use of child soldiers, 2010; Elbert et al., 2010; Schauer & Elbert, 2010; United Nations, 2007), we made the following predictions: as a consequence of the exposure to extreme forms of violence, we predicted that: (1) former child soldiers show a greater PTSD symptom severity than adult combatants. Furthermore, in comparison to adult combatants, we hypothesized that: (2) former child soldiers would report having perpetrated more different types of violence; and (3) show more appetitive aggression. To gain more knowledge about the characteristics of former child soldiers, we additionally examined the relationship of their mental health status to their experiences in armed groups.

## Methods

### Sample

All interviews were conducted in Goma, DRC. Most interviews, 72% (*n*=162), took place at a UN demobilization transit camp, 27% (*n*=60) were conducted at a reintegration center for former child soldiers and former combatants and 1% (*n*=2) at a military detention facility. All combatants who demobilize in the province of North Kivu, pass through the UN demobilization camp in Goma. All combatants who were registered in the demobilization camp during the time of our assessment participated in the study. The reintegration center for former child soldiers and combatants was led by a Congolese non-governmental, non-profit organization and offered vocational training in manual trades. All former child soldiers and combatants enrolled in the program took part in the interviews. Time since demobilization ranged from 1 day to 7 years. The majority of the sample (79%) demobilized within the year prior to assessment.

Out of a full sample of 224 interviews, 11 could not be completed for logistical reasons. Additionally, 13 participants without combat experience were excluded from further analysis. The remaining sample was completely male. A variety of armies and armed groups were represented, but, in most cases, the combatants belonged to the FARDC, CNDP, FDLR or to one of several Mai-Mai groups.

Out of the remaining *N*=200 interviews, we identified all participants that joined the first armed group below the age of 18 years as former child soldiers. Thus, the group consisted of *n*=126 with a mean age at recruitment of 13.20 years (SD = 3.07, range: birth–17). The age at the time of assessment was 20.72 years (SD = 3.65, range: 15–32). The majority (79%, *n*=99) was born in the DRC, and 21% were born in Rwanda (*n*=27).

Participants who joined the first armed group with an age of at least 18 years were identified as adult ex-combatants. The group consisted of *n*=74 ex-combatants, with a mean age at recruitment of 23.11 years (SD = 4.49, range: 18–37) and a mean age at the time of assessment of 30.97 years (SD = 6.76, range: 19–50). The majority was born in the DRC (68%, *n*=50), a minority was born in Rwanda (31%, *n*=23) and one participant was born in Uganda.

### Measures

All instruments were applied as a semi-structured interview and the same interview set was used in all interview settings. The clinical experience of the interviewers and the administration of the instruments in interview form allowed the interviewers to use the same questions for adults and minors.

#### Socio-demographic data and military experiences

The first part of the interview involved collecting information about the interviewee's age, place of birth, and level of education. Additionally, we asked about the time within armed groups, combat experience, the highest military rank, number of enlistments in the same or different armed groups, and the recruitment type. We defined enlistment as entry into an armed group. Many former combatants and child soldiers joined again an armed group in their past after a failed attempt to integrate into civil society. We assessed these failed attempts through reenlistment as we counted the number of enlistments to armed groups in their past. We assessed the recruitment type (voluntary vs. forcibly recruited) by counting the interviewee's subjective perception of his recruitment.

#### Violence types

To assess the number of lifetime experienced violence types, a list of war- and non-war-related potentially traumatic events was adapted to the circumstances of armed groups. The list included events from the checklist of traumatic events of the Posttraumatic Stress Diagnostic Scale (Foa, Cashman, Jaycox, & Perry, 1997) and was closely related to a checklist that previously demonstrated a high test–retest reliability (*r*=0.73, *p*<0.001), significant accordance with the CIDI Event List (Ertl et al., 2010) and a significant correlation with cortisol (Steudte et al., 2011) in a study with Ugandan child soldiers. The list consisted of 31 event types, for example, domestic violence, assault by weapon, rape, accidents, and massacres. The number of times a specific event had been experienced was not assessed, as distorted memory in PTSD renders this measure unreliable (Elbert & Schauer, 2002; Kolassa & Elbert, 2007; McNally, 2006). For the analysis, we computed a score of experienced violence types (range 0–7), including, for example, being physically or sexually assaulted, as well as a score perpetrated violence types (range 0–9) including, for example, assaulting someone else physically or sexually. For this study, Cronbach's *α* was 0.74.

#### Mental health

Symptom severity of PTSD was assessed with the help of the PTSD Symptom Scale-Interview (PSS-I) (Foa, Riggs, Dancu, & Rothbaum, 1993). It consists of 17 items, whereas each item corresponds to one PTSD symptom as specified in DSM-IV with a range from *not at all or only one time* (0) to *five times per week or more/almost always* (3) concerning the past 4 weeks. The PSS-I comes with reliable psychometric properties (Foa & Tolin, 2000) and was validated in the Great Lakes region by Ertl et al. (2010). The Cronbach's *α* coefficient was 0.86 and the inter-rater reliability 0.93 for the PSS-I sum score (Foa et al., 1993; Foa & Tolin, 2000). This study used the PSS-I score, which ranges from 0 to 51. For this study, Cronbach's *α* was 0.90.

Appetitive aggression was assessed with the 15-item Appetitive Aggression Scale (AAS) (Weierstall & Elbert, 2011), which has been validated with over 1,600 ex-combatants and child soldiers and proven its good psychometric properties in comparable samples (Weierstall et al., 2011, 2012). A question regarding the perception of violence or appetitive aggression was given to the interviewee in each item (e.g., *Is it exciting for you if you make an opponent really suffer?; Once fighting has started, do you get carried away by the violence?; When you fight, do you stop caring about whether you could be killed?*). The interviewee rated the level of agreement with the given question on a five-point Likert scale ranging from *disagree (0)* to *agree (4)*. For the analyses, a sum score of all 15 items was computed, ranging from 0 to 60. For this study, Cronbach's *α* was 0.89.

### Procedure

All participants gave their informed consent verbally. In addition, the respective institutions gave their informed consent for underage participants, due to the fact that their caregivers were either dead or not available. The ethical review board of the University of Konstanz as well as the United Nations’ mission in the DRC (MONUSCO) and authorities of the reintegration center approved this study. Other parts and aspects of the data gathered during the extensive investigation are presented in other recent reports (Hecker et al., 2012; Hecker, Hermenau, Maedl, Schauer et al., 2013; Hecker, Hermenau, Maedl, Hinkel et al., 2013).

Four psychologists and a nurse, each having had extensive work experience in East Africa, conducted the interviews with the help of three interpreters. The interpreters were trained in the concepts of mental disorders and aggression before the assessment. All instruments were translated into Kiswahili, Kinyarwanda, or Lingala, and the translation was intensely discussed to guarantee a precise interpretation. Two of the interviewers could speak the native languages fluently and continuously supervised and assured valid translation. The interviewers had standardized the form of assessment by practicing in joint interviews. Subsequently, one interviewer and one interpreter individually interviewed each participant in a calm and private setting. The interview took on average one and a half hours. All participants received 2 US dollars for participating. We assured mental hygiene of the interview team through daily intervision and supervision.

### Analyses

For each set of variables, namely military experiences (type of recruitment, time with armed groups, military rank, number of enlistments in armed groups), mental health (PTSD, appetitive aggression), and reported violence (experienced and perpetrated violence), a Multivariate Analysis of Variance (MANOVA) was conducted. Kurtosis was between *K*= − 2.01 and 1.49 and skewness was between *S*= − 0.68 and 1.42. Thus, no variable deviated from normal distribution. No univariate or multivariate outliers were detected. Box-M-Test for homogeneity of variance–covariance matrices produces *F*(10, 110067) = 2.20, *p*=0.015 for military experience variables, *F*(3, 807079) = 3.14, *p*=0.024 for reported violence variables, and *F*(3, 807079) = 1.59, *p*=0.190 for mental health variables. As Box-M-Test is a very sensitive test, we adjusted the *α*-level to *α*=0.01. Consequently, variance–covariance matrices did not deviate significantly from homogeneity. Subsequently, we performed a Roy–Bargmann Stepdown Analysis on the dependent variables to investigate the contribution of each variable. To test the relations between mental health variables and experiences of former child soldiers, we used the Pearson coefficient of correlation. All analyses used a two-tailed *α*=0.05, unless otherwise specified. In cases of multiple testing, we adjusted the *α*-level using a Bonferroni correction to avoid *α*-inflation. Our metric for a small effect size was *η* ^2^=0.01, for a medium effect, *η* ^2^=0.06; and for a large effect; *η* ^2^=0.14.

## Results

### Descriptive results

Almost all former child soldiers reported at least one experienced violence type (100%, *n*=126) and at least one perpetrated violence type (99%, *n*=125). Concerning posttraumatic stress symptoms, 14% (*n*=17) reported no symptoms at all. In total, 29% (*n*=37) of the former child soldiers fulfilled the DSM-IV criteria for a PTSD diagnosis. Only a minority of 2% (*n*=2) reported no appetitive aggression at all. Almost all former adult combatants reported to have experienced at least one violence type (100%, *n*=74) and to have perpetrated at least one violence type (97%, *n*=72). Additionally, 27% (*n*=20) reported no posttraumatic symptoms, and 7% (*n*=5) reported no appetitive aggression. Of all adult ex-combatants, 16% (*n =*12) fulfilled a PTSD diagnosis according to DSM-IV. Means and frequencies of both groups concerning their time in armed groups, experienced and perpetrated violence types, as well as posttraumatic stress symptom severity and appetitive aggression are detailed in Table 1.

**Table 1:** Descriptive data concerning military experiences, mental health and violence in former child soldiers and adult combatants

	Child soldiers (*n*=126)		Adult combatants (*n*=74)	
		
	M or *n*	SD or %	M or *n*	SD or %
Recruitment type				
Voluntary	66	52	39	53
Forcibly	60	48	35	47
Time with armed groups (in weeks)	329	232	400	324
Military rank				
Holding a rank	43	34	45	61
Holding no rank	83	66	29	39
Number of enlistments in armed groups	1.50	0.70	1.60	0.89
AAS score	27.15	13.25	19.61	14.29
PSS-I score	11.55	9.33	7.64	7.65
Perpetrated violence types	5.38	1.81	4.22	1.84
Experienced violence types	4.60	0.92	3.76	1.19

M = mean, SD = standard deviation.

### Military experiences

A MANOVA revealed that at least one of the military experience variables, including type of recruitment, time with armed groups, military rank, and number of enlistments in armed groups, differed between former child soldiers and former adult combatants (*F*(4, 195) = 4.20, *p*=0.003, *η* ^2^=0.08). A Roy–Bargmann Stepdown Analysis was performed on an *α*-level of *α*=0.017 due to Bonferroni correction. The recruitment type (voluntary vs. forcibly; stepdown *F*(1, 198) < 0.01, *p*=0.965, *η* ^2^<0.01) and the time with armed groups (stepdown *F*(1,197) = 3.39, *p*=0.067, *η* ^2^=0.02) did not differ between former child soldiers and former adult combatants. With differences due to recruitment type and time with armed groups already entered, holding a military rank differed between former child soldiers and former adult combatants (stepdown *F*(1, 196) = 13.00, *p*<0.001, *η* ^2^=0.06). As shown in Table 1, former adult combatants more often occupied military ranks than former child soldiers. The number of enlistments in armed groups does not differ significantly between former child soldiers and former combatants (stepdown *F*(1, 195) = 0.27, *p*=0.601, *η* ^2^<0.01).

### Violence

A MANOVA showed that at least one of the variables of reported violence types differed significantly between child soldiers and adult combatants (*F*(2, 197) = 17.44, *p*<0.001, *η* ^2^=0.15). A Roy–Bargmann Stepdown Analysis was performed on an *α*-level of *α*=0.025 due to Bonferroni correction. The reported perpetrated violence types made a unique contribution of *η* ^2^=0.09 (stepdown *F*(1, 198) = 18.89, *p <*0.001). The former child soldiers reported more perpetrated violence types than the former adult combatants (see Table 1). With differences due to the perpetrated violence types already entered, experienced violence types made a unique contribution of *η* ^2^=0.07 (stepdown *F*(1, 197) = 14.68, *p <*0.001). In detail, the former child soldiers reported more experienced violence types than the former adult combatants (see Table 1).

### Mental health: PTSD and appetitive aggression

A MANOVA revealed that at least one of the mental health variables, including PSS-I score and AAS score, differed between former child soldiers and former adult combatants (*F*(2, 197) = 10.97, *p <*0.001, *η* ^2^=0.10). A Roy–Bargmann Stepdown Analysis was performed on an *α*-level of *α =*0.025 due to Bonferroni correction. Both variables differed significantly between the two groups. The contribution made by the PSS-I score was *η* ^2^=0.05 (stepdown *F*(1, 198) = 9.33, *p =*0.003). Former child soldiers showed higher PSS-I scores than former adult combatants (see Table 1). With differences due to the PSS-I score already entered, the AAS score of appetitive aggression showed a unique contribution of *η* ^2^=0.06 (stepdown *F*(1, 197) = 12.08, *p =*0.001). Former child soldiers reported higher appetitive aggression than former adult combatants (see Table 1).

To test the relationship between AAS and experiences in armed groups of former child soldiers an adjusted *α*-level of *α =*0.010 due to Bonferroni correction was used. All correlations are displayed in Table 2. In former child soldiers, the AAS score correlated positively with holding a military rank, the number of enlistments in armed groups and voluntary recruitment. Furthermore, the AAS score correlated positively with perpetrated violence types in former child soldiers, but not with experienced violence types. In contrast, the PSS-I score correlated positively with experienced violence types, but not with perpetrated violence types (see Table 2). The PSS-I score did not correlate significantly with any of the military experience variables using again an adjusted *α*-level of *α =*0.010 due to Bonferroni correction. Moreover, AAS and PSS-I score did not correlate significantly.

**Table 2:** Inter-correlations of relevant variables in former child soldiers

	AAS	PSS-I	RT	TAG	MR	NE	PVT	EVT
AAS score	1							
PSS-I score	0.03	1						
Recruitment type (RT)	0.26*	−0.11	1					
Time with armed groups (TAG)	0.12	0.10	0.25*	1				
Military rank (MR)	0.27*	−0.02	0.12	0.12	1			
Number of enlistments (NE)	0.27*	−0.03	0.14	0.06	0.37**	1		
Perpetrated violence types (PVT)	0.46**	0.14	0.07	−0.06	0.20	0.07	1	
Experienced violence types (EVT)	0.10	0.23*	−0.11	−0.14	0.08	−0.03	0.35**	1

* *p*≤0.01

** *p*≤0.001.

## Discussion

Experiences of war and armed conflict can lead to severe mental suffering and illness in combatants, especially when the individual joined the armed group as a minor. Exposure to violence and perpetration of violent acts can lead to mental health problems like posttraumatic stress symptoms and aggressive behavior (Maclure & Denov, 2006; Schauer & Elbert, 2010).

According to our results, former child soldiers can be characterized as follows: while they resembled adult combatants concerning the recruitment type and time within armed groups, former child soldiers were less likely to hold military ranks than former adult combatants. Consistent with the literature (Schauer & Elbert, 2010), former child soldiers reported being both victims and perpetrators of violence. For example, they reported more experienced and perpetrated violence types than adult former combatants. Consequently, former child soldiers also showed higher PTSD symptom severity and higher appetitive aggression than former adult combatants. These findings are in accordance with other research reporting that especially child soldiers suffer from trauma-related disorders and aggressive behavior (Betancourt et al., 2010; Derluyn et al., 2004; Schauer & Elbert, 2010). Moreover, the results revealed that exposure to violence is linked to higher PTSD symptom severity in former child soldiers. Thus, our results confirmed the building block effect in child soldiers: Repeated exposure to different types of traumatic stressors cumulatively heightens the risk to develop PTSD symptoms (Neuner et al., 2004). In summary, this study has shown that former child soldiers reported more adverse experiences during service and more mental health problems after demobilization than adult former combatants. We found no direct relationship between appetitive aggression and PTSD, which is in line with recent findings (Hecker, Hermenau, Maedl, Schauer, et al., 2013; Weierstall et al., 2013) showing that the protective effect of appetitive aggression wanes if the level of traumatization is high.

Additionally, we found a positive relationship between appetitive aggression and perpetrated violence types in former child soldiers. Furthermore, the positive correlation between appetitive aggression and military rank indicates that former child soldiers, who perceive perpetrating violence as fascinating and arousing, seem more likely to be promoted in the military hierarchy of an armed group. This finding is consistent with a study of former child soldiers in Uganda (Crombach et al., 2013). Concordantly, other studies reported a gradual transformation in the perception of the perpetration of violence in child soldiers, who were forced to perpetrate violence: At first it was frightening, however, with repeated experience it became not only normal and acceptable, but even exciting and arousing (Elbert et al., 2010; Maclure & Denov, 2006). Thus, living in an extremely violent environment such as in armed groups may reinforce the appetitive perception of aggression and violence in former child soldiers and in this way increase the perpetration of violence. Furthermore, appetitive aggression was positively related to voluntary recruitment in former child soldiers. Highly appetitively aggressive child soldiers tend to perceive their recruitment as voluntarily more often than low appetitively aggressive child soldiers. However, this study cannot determine whether child soldiers showed appetitive aggression already before their enlistment or whether they developed appetitive aggression during their time with an armed group. Therefore, longitudinal studies are highly important to understand the development of appetitive aggression in child soldiers and its causal relationship to perpetrated violence.

Although we did not investigate reintegration in detail in this study, we can conclude from prior findings that suffering from PTSD and aggression can lead to discontinuation of reintegration programs and consequently heighten the risk of voluntary reenlistment in armed groups (Betancourt et al., 2008; Boyden, 2003; Mogapi, 2004; Stott, 2009). Similarly, we found that former child soldiers with higher appetitive aggression rejoined armed groups more often. Appetitive aggression therefore may interfere with the success of reintegration programs and heighten the risk of voluntary reenlistment in armed groups. Former child soldiers who behave aggressively are at high risk of failing in reintegration programs. As prospects in a conflict region like the eastern DRC are limited, they are more likely to go back to military life and armed conflict. As most participants in this study very recently left the armed groups and were still in the process of integration, we only focused on one variable concerning reintegration and could not include typical integration variables like employment or socio-economic status. Future studies should investigate the possible challenges appetitive aggression poses to integration more closely. Moreover, future research should include further psychological reactions to traumatic stress like depression, anxiety, and guilt and their impact on integration.

This study demonstrates that former child soldiers are burdened in two ways: suffering from PTSD and displaying appetitively aggressive behavior. To address their needs and ease their suffering, we advocate adding a mental health component to reintegration programs for former child soldiers. If a young man is suffering from PTSD or displaying an enhanced readiness for aggression, his ability to profit from reintegration programs, such as vocational training in manual trades will be severely impaired (Betancourt et al., 2008; Boyden, 2003). However, mental suffering and aggression have not been in the focus of most reintegration programs so far (Maedl et al., 2010; Medeiros, 2007; Mogapi, 2004; Stott, 2009). It is imperative to redress these deficits by adding a psychological/psychotherapeutic intervention to the established economic and social reintegration programs (Betancourt et al., 2008; Hermenau, Hecker, Schaal, Maedl, & Elbert, 2013; Mogapi, 2004; Stott, 2009).

Some methodological aspects limit the generalization of these findings. The cross-sectional study design and the specific sample do not allow us to establish causality. While certainly representation of other settings cannot be claimed, the consistency with findings from other countries concerning child soldiers, mental suffering and appetitive aggression demonstrates that similar relationships would also be found in other settings. Although we interviewed all of the former child soldiers and adult combatants who were enrolled in the programs of the United Nation and the reintegration center during the assessment period, the sample might not be representative for child soldiers and adult combatants in the eastern DRC. In general, most studies include only former combatants and no active combatants. This may lead to an incomplete picture and selection bias. Even though we practiced in joint interviews to standardize the assessment, we cannot entirely rule out influences of interviewers. Moreover, differences in interview settings as well as the membership of different armed groups might have influenced the results. The on-going conflict in eastern DRC and the large and changing variety of armed groups in DRC made it practically impossible to control for the membership of armed groups. Furthermore, differences in current age might have influenced the results. Although the majority of our sample was adult at the time of assessment, some minors were included in the group of child soldiers. Unfortunately, we were not able to include a comparison group with the same age as the child soldiers. However, we think that the influences of current age, age at time of recruitment, the time with armed groups, and the time since leaving the armed group are very difficult to disentangle. As we focused on child soldiers and combatants who recently left the armed group and spent a comparable time with armed groups, we had to accept an age difference. All in all, the former child soldiers and combatants talked very openly about their experiences and mental health. However, a potential bias, such as social desirability, can never be ruled out for subjective reports.

## Conclusion

This study closely investigated the characteristics and experiences of former child soldiers in the on-going conflict in the DRC. Former child soldiers suffered from being both perpetrators and victims of violent acts. Compared with a group of adult former combatants, former child soldiers presented a higher severity of trauma-related symptoms. They also reported perceiving perpetrating violence as more fascinating and arousing. Being more appetitively aggressive was related to higher positions in armed groups and more perpetrated violence types. Additionally, high appetitive aggression was linked to repeated reenlistment in armed groups. Thus, our findings indicate that growing up in an armed group is linked to higher levels of trauma-related disorders, aggressive behavior, and failed reintegration. Consequently, particularly former child soldiers burdened with trauma-related illness and substantial appetitive aggression pose challenges for successful integration into civil society. It is thereby imperative to consider the mental health issues of former child soldiers when designing adequate reintegration programs. As the demobilization and reintegration of former child soldiers remains an important piece of the puzzle when solving on-going conflicts, it is crucial to specifically address mental suffering and enhanced aggressive behavior of former child soldiers to help them find closure with their past and start a new life in civil society.
